# Effect of a Qigong Intervention on Telomerase Activity and Mental Health in Chinese Women Survivors of Intimate Partner Violence: A Randomized Clinical Trial

**DOI:** 10.1001/jamanetworkopen.2018.6967

**Published:** 2019-01-11

**Authors:** Denise Shuk Ting Cheung, Wen Deng, Sai-Wah Tsao, Rainbow Tin Hung Ho, Cecilia Lai Wan Chan, Daniel Yee Tak Fong, Pui Hing Chau, Athena Wai Lin Hong, Helina Yin King Yuk Fung, Joyce Lai Chong Ma, Agnes F. Y. Tiwari

**Affiliations:** 1 School of Nursing, Li Ka Shing Faculty of Medicine, University of Hong Kong, Hong Kong, China; 2 School of Biomedical Sciences, Li Ka Shing Faculty of Medicine, University of Hong Kong, Hong Kong, China; 3 Centre on Behavioral Health, The University of Hong Kong, Hong Kong, China; 4 Department of Social Work and Social Administration, University of Hong Kong, Pokfulam, Hong Kong, China; 5 Hong Kong Sheng Kung Hui Lady MacLehose Centre, Kwai Chung, New Territories, Hong Kong, China; 6 Department of Social Work, Chinese University of Hong Kong, United College, Shatin, New Territories, Hong Kong, China; 7 School of Nursing, Hong Kong Sanatorium and Hospital, Wong Chuk Hang, Hong Kong, China

## Abstract

**Importance:**

Qigong is a mind-body exercise that may be an effective self-care intervention for improving the well-being of women survivors of intimate partner violence.

**Objective:**

To test whether a qigong intervention would increase telomerase activity and improve mental health in Chinese women who survived intimate partner violence.

**Design, Setting, and Participants:**

A single-blind randomized clinical trial among Chinese women (N = 271) who survived intimate partner violence in the past 2 years recruited from a community center in Hong Kong, China. The trial was conducted from March 12, 2014, to May 26, 2016. Data analysis was by intention to treat and performed from June 7 to August 24, 2018.

**Interventions:**

Randomization (1:1) to a 22-week qigong intervention (n = 136) that included 22 weeks of Baduanjin qigong group training (1-6 weeks: 2-hour sessions biweekly; 7-22 weeks: 1-hour follow-up sessions weekly) and self-practice (30 minutes per day for 22 weeks) or to a wait-list control group (n = 135) that received optional monthly health education sessions unrelated to qigong after 6 weeks (posttraining period) and qigong training after 22 weeks (postintervention period).

**Main Outcomes and Measures:**

The primary outcome was telomerase activity in peripheral blood mononuclear cells. The secondary outcomes included levels of proinflammatory cytokines (tumor necrosis factor and interleukin 6) in peripheral blood plasma, depressive symptoms (Beck Depression Inventory II score; score range, 0-63; higher scores represent more severe depressive symptoms), perceived stress (Perceived Stress Scale; score range, 0-40; higher scores represent higher stress), and perceived coping (Perceived Coping Scale; score range, 0-13; higher scores represent use of more coping strategies).

**Results:**

From 1611 Chinese women screened (mean [SD] age, 42.0 [8.8] years), 247 of 271 randomized participants completed the study (intervention group, 120; wait-list control group, 127). Telomerase activity of the intervention group participants after 22 weeks was not significantly different from that of the wait-list control group participants (5.18 U [95% CI, 5.05-5.31 U] in the intervention group vs 5.14 U [95% CI, 5.01-5.27 U] in the wait-list control group; *P* = .66). The mean change in telomerase activity from baseline was marginally significant in the intervention group (effect size [*d*], 0.13; 95% CI, 0.001-0.27) but not in the wait-list control group (*d*, −0.03; 95% CI, −0.16 to 0.10). Perceived stress and depressive symptoms were significantly lower in the intervention group than in the wait-list control group after 6 weeks (between-group differences: perceived stress: *d*,  −1.81; 95% CI, −3.27 to −0.34; depressive symptoms: *d*,  −3.57; 95% CI, −6.25 to −0.90), but not after 22 weeks (between-group differences: perceived stress: *d*, −1.03; 95% CI, −2.50 to 0.43; depressive symptoms: *d*,  −1.78; 95% CI, −4.26 to 0.70).

**Conclusions and Relevance:**

The findings of this study do not support a significant benefit of Baduanjin qigong on telomerase activity in women who have survived intimate partner violence. However, outcomes related to mental health seem to be improved, which should be confirmed by additional studies.

**Trial Registration:**

ClinicalTrials.gov Identifier: NCT02060123

## Key Points

**Question:**

What is the effect of a qigong intervention on telomerase activity in Chinese women survivors of intimate partner violence?

**Findings:**

In this randomized clinical trial that included 271 Chinese women survivors of intimate partner violence, the between-group difference in telomerase activity after 22 weeks was not statistically significant.

**Meaning:**

The qigong intervention did not have a significant benefit on telomerase activity in Chinese women survivors of intimate partner violence.

## Teaser

This randomized clinical trial examines the effect of a qigong intervention on telomerase activity in cells and mental health in women survivors of intimate partner violence in China.

## Introduction

Intimate partner violence (IPV) against women is a serious, pervasive, global public health problem.^1^ Despite the abundant published findings linking IPV to negative health outcomes,^2^ the underlying mechanism remains unclear. It is often assumed that living in an atmosphere of fear, coercion, and shame may render survivors of IPV more susceptible to chronic psychological stress.^3^

The latter is thought to hasten cellular aging as evidenced by accelerated telomere shortening.^4,5,6^ Telomeres are DNA-protein complexes that cap and protect chromosomal ends from degradation or fusion.^7^ When telomeres shorten to a critical point, cells will enter senescence. Organ dysfunction may ensue owing to excessive accumulation of senescent cells. Previously, a link between exposure to IPV or duration of IPV-related stress and telomere shortening was found among abused women.^8^ Because accelerated cellular aging may attenuate normal bodily functions and lead to greater morbidity,^7^ this phenomenon may explain the greater morbidity among the survivors of IPV.

Telomerase is an enzyme that elongates telomeres and functions to promote cell longevity.^7,9^ Telomerase has multiple extratelomeric functions, including enhancing stress resistance and blocking apoptosis,^10^ which play important roles in the antiaging processes. Previous studies have suggested that telomerase activity may be improved through health-promoting behaviors (eg, intensive meditation training^11^ and comprehensive lifestyle changes).^12^ Qigong, a mind-body intervention, has also been suggested as a holistic health practice to enhance telomerase activity^13^ and to promote physical and mental well-being.^14^ In the context of IPV, there is scanty evidence on how the effects of psychological stress on cellular aging may be reduced. The purpose of this randomized clinical trial was to test whether qigong would increase telomerase activity and improve mental health in Chinese women survivors of IPV.

## Methods

### Trial Design, Setting, and Participants

This 22-week, parallel randomized clinical trial (RCT) comparing a qigong intervention with a wait-list control was conducted from March 12, 2014, to May 26, 2016. Reporting in this study followed the Consolidated Standards of Reporting Trials (CONSORT) reporting guideline. Ethical approval was obtained from the institutional review board of the University of Hong Kong and Hospital Authority Hong Kong West Cluster. The trial protocol (Supplement 1) has been previously reported^15^ and was followed, except that the amendment was made to the statistical analysis plan at the time of this article’s preparation. The participants were recruited between March 12, 2014, and October 25, 2015, from a large community center that provides diversified health and social services for users of all age groups in 3 districts in Hong Kong, covering a population of approximately 830 000 individuals. All the participants provided written informed consent. Flyers promoting the study were posted on the notice boards of outreach sites of the community center. Interested individuals would contact center staff for further screening. Recruitment was also publicized by center staff.

Chinese women were eligible to participate if they were 18 years or older, willing to undertake the qigong intervention, available for all the data collection points, and receptive to random allocation and had survived IPV in the preceding 2 years. Women were excluded if they had participated in qigong training or another mind-body intervention within the previous 6 months or had serious medical conditions that might limit their participation in qigong. They were also excluded if they had psychiatric disorders, used medication or a psychological intervention for stress, or were abused by someone who was not their intimate partner based on their self-reports. To confirm a woman’s eligibility, a screening in the form of a face-to-face interview was conducted by a center-based social worker in a private area without the presence of her partner. The social worker responsible for screening was not involved in subsequent data collection.

### Randomization and Blinding

Eligible participants were randomized to either the intervention or the wait-list control group, assigned at a 1:1 ratio by block randomization with randomly selected block sizes of 4, 6, and 8. The list was computer generated, recorded by an investigator (D.S.T.C.) who was not involved in subject recruitment, and placed in numbered, sequential, sealed, opaque envelopes. The envelopes were kept by a center staff person who was not involved in the study; thus, the randomization was centrally controlled to avoid any bias in selection. Outcome assessors and research assistants who entered data were not involved in the study design, did not know the study hypotheses, and were blinded to group assignment. The nurse who collected the blood and the laboratory technicians who processed the blood samples did not know which group the participant and blood sample belonged to and were blinded to the group assignment. Participants were not blinded to group allocation. To minimize the possible effects from knowledge of group assignment on self-reported outcomes of the participants, the participants were told they had to be randomized into 2 groups with different sequences for receiving qigong training because of limited class size.

### Intervention and Control Conditions

One of the various forms of qigong is Baduanjin, meaning “8 pieces of silken brocade” in Mandarin. It consists of 8 movements that are performed in a smooth and graceful manner, hence the name. The movements, combined with breathing and meditation, exercise the mind and body for healing. Baduanjin was selected as the intervention because it has been standardized by the Chinese Health Qigong Association^16^ and is easy to perform.

The intervention lasted 22 weeks and was composed of (1) group training (12 two-hour qigong sessions for 6 weeks [twice weekly]); (2) weekly group follow-up (16 one-hour qigong follow-up practice sessions from week 7 to week 22 [once weekly]); and (3) self-practice (participants were encouraged to practice qigong 30 minutes per day for the entire intervention period [22 weeks]). The sessions were delivered by a certified qigong master with more than 20 years of experience in teaching qigong, who was provided with standardized content of training and follow-up sessions. For each of the training sessions, the master first briefly introduced the basic theories of mind-body connections and then led the group to perform gentle body movement to facilitate a free flow of qi through the energy channels. This warm-up was followed by Baduanjin training. For weekly follow-up sessions, the master practiced Baduanjin with the participants, with the aim of providing reinforcement of learning and remedial teaching. A research assistant was assigned to each session to monitor the attendance of participants, their adherence to the intervention, and the qigong master’s use of standardized elements. To monitor the self-practice of qigong in the intervention group, each participant entered the details of duration and frequency of qigong undertaken every day into a record card, which was submitted to the research assistant weekly.

Participants in the wait-list control group could choose to attend monthly health education sessions unrelated to qigong and given by a registered nurse after 6 weeks on the wait list and to receive postintervention qigong training after data collection was completed (after 22 weeks).

### Data Collection

Data were collected at baseline, after training (after 6 weeks [ie, on completion of the 6-week group training]), and after the intervention (after 22 weeks [ie, on completion of the entire qigong intervention]). A blood sample was collected from each participant by a registered nurse at baseline and after 22 weeks for measurements of telomerase activity and proinflammatory cytokines. Questionnaire measures including the Revised Conflict Tactics Scales (CTS2), Perceived Stress Scale (PSS), Beck Depression Inventory II (BDI-II), and Perceived Coping Scale^17^ (PCS) were administered to participants of both groups at the baseline, posttraining, and postintervention assessments. The Abuse Assessment Screen (AAS) and demographic questionnaire were administered only at baseline, whereas the study evaluation questionnaire was administered only at the postintervention assessment.

### Outcome Measures

The primary outcome measure was telomerase activity in peripheral blood mononuclear cells. Secondary outcome measures included levels of proinflammatory cytokines (tumor necrosis factor [TNF] and interleukin [IL] 6) in peripheral blood plasma, depressive symptoms, perceived stress, and perceived coping.

### Biomarker Assessment

For measurements of telomerase activity in peripheral blood mononuclear cells and proinflammatory cytokines in peripheral blood plasma, 10 mL of peripheral blood was collected from each participant at baseline and at postintervention assessment. The peripheral blood mononuclear cells and plasma were separated. Telomerase activity was analyzed using an enzyme-linked immunosorbent assay (ELISA) kit (TeloTAGGG Telomerase PCR ELISA^PLUS^; Roche) and was quantified as relative telomerase activity against a low-activity internal standard supplied with the kit. The levels of TNF and IL-6 (ie, proinflammatory cytokines that serve as biochemical modulators of telomerase activity and are closely related to chronic psychological stress)^12^ were measured with ELISA kits (Bio-Station Limited) as exploratory analyses.

### Questionnaire Measures

The validated 5-item Chinese version of the AAS^18^ was used to screen potential participants for IPV. A woman had a positive screening result for IPV if she answered yes regarding whether she experienced emotional, physical, or sexual abuse by her former or current intimate partner in the preceding 2 years. The validated Chinese version of the CTS2,^19^ rated on an 8-point Likert scale, was used to evaluate the type and frequency of abusive behaviors by the partner during conflicts in the past year and, if not, if they ever occurred prior to the past year. The CTS2 is composed of 27 items on psychological aggression, physical assault, and sexual coercion. Scores range from 0 to 675, and higher scores indicate higher frequency of abusive acts. The validated Chinese version of the 10-item PSS,^20^ rated on a 4-point Likert scale, was used for measuring perception of stress during the past month. Respondents have to rate the degree to which life situations are perceived as stressful. Scores on the PSS range from 0 to 40, and higher scores represent higher stress. The validated Chinese version of the 21-item BDI-II,^21^ rated on a 4-point Likert scale, was used to evaluate depressive symptoms in the previous 2 weeks. It quantifies cognitive, affective, and somatic components of depression. Scores on the BDI-II range from 0 to 63, and higher scores indicate more severe depressive symptoms. The validated Chinese version of the 14-item PCS^22^ was administered to assess use of coping strategies by the participant in dealing with relationship conflicts. Scores on the PCS range from 0 to 13, and higher scores represent use of more coping strategies. The demographic questionnaire (19 items) and study evaluation questionnaire (8 items) were designed by the research team to elicit demographic information and feedback on the study, respectively. In the study evaluation questionnaire, intervention group participants rated how much they liked or disliked practicing qigong on a 4-point Likert scale, whereas the wait-list control group participants indicated whether they practiced qigong during the study period.

### Sample Size

Sample size calculation was based on the primary comparison of telomerase activity between the intervention and wait-list control groups. We anticipated approximately 0.05 to 0.1 U (mean, 0.075 U) of improvement (an increase by 75%) in telomerase activity in the qigong group with an SD of 0.2, based on a previous study.^23^ Of note, an increase in telomerase activity of approximately 75% in caregivers was associated with an extension of telomere length, which was regarded as having clinically significant implications for human health.^6^ To detect at least 0.075-U differences between the 2 groups with 80% power and at most a 5% chance of committing a false-positive error, we needed 113 participants per group. Allowing for attrition of 5% (based on a previous clinical trial^24^ held in the same community where the present study was conducted), we rounded up the sample size to at least 240 participants in total.

### Data Analysis

Data analysis was performed from June 7 to August 24, 2018, in SPSS Statistics 20 software (IBM) via the intention-to-treat approach. Baseline characteristics between the intervention and wait-list control groups were compared by the χ^2^ test and Mann-Whitney test for categorical and continuous data, respectively. Mixed-effects models were used to estimate the effect size (*d*) at week 6 (except biomarkers) and week 22, with 95% CI, in terms of (1) between-group differences (primary analysis), (2) within-group change from baseline, and (3) between-group difference in change from baseline in the outcomes. Time, group, and interaction between group and time were included as independent variables. Bonferroni adjustment was used for multiple pairwise comparisons. Probability plots were constructed for assessing normality of residuals. A 5% level of significance was assumed, and all significance tests were 2-sided. The initial statistical analysis plan can be found in the trial protocol in Supplement 1 and was modified. The current analysis did not (1) adjust baseline values owing to absence of significant difference in baseline values; (2) use regression models for biomarker analysis owing to the use of a more sophisticated statistical method (ie, mixed-effects model); and (3) replace missing values by the last observed values because the mixed-effects model can accommodate missing data and does not require imputation of missing observations, providing a natural way to deal with missing values or dropouts.^25^

## Results

### Participants

Of the 1611 Chinese women screened (mean [SD] age, 42.0 [8.8] years), 271 women were randomized into either the intervention (n = 136) or wait-list control (n = 135) group (Figure). Data collection was completed for 120 women in the intervention group (88.2%) and 127 women in the wait-list control group (94.1%). Overall, 24 women (8.9%) withdrew from the study; the major reasons for withdrawal were a lack of time or loss of interest. Women in the intervention group who completed the study (n = 120) self-practiced qigong for a mean (SD) 103.3 (138.2) minutes per week throughout the intervention. Most of them (89 [74.2%]) attended 80% or more of the training sessions. There were no reports of adverse events or harm arising from participation in the study.

**Figure.**
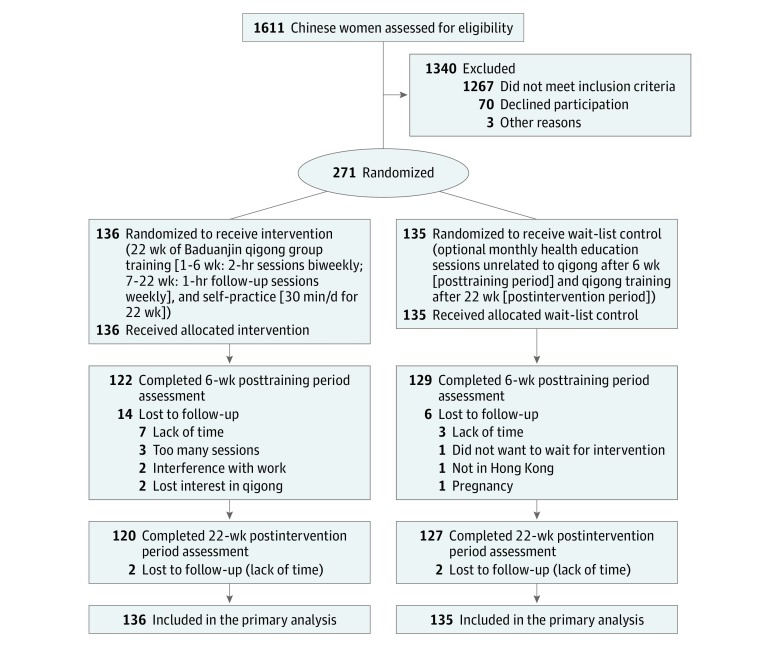
Flow of Participants Through the Study

Log transformation was performed for the biomarker data to improve normality as suggested by the probability plots. Table 1 provides information on the demographic characteristics of the participants. The mean (SD) age in the intervention group was 42.0 (8.7) years and in the wait-list control group was 41.5 (9.3) years. The mean (SD) number of years of marriage was 21.3 (23.4) in the intervention group and 20.2 (21.2) in the wait-list control group. At baseline, there were no statistically significant differences in the participants’ sociodemographic characteristics or outcome measures between the intervention and wait-list control groups.

**Table 1.** Baseline Sociodemographic Characteristics Among Chinese Women Survivors of Intimate Partner Violence Receiving a Qigong Training Intervention vs Wait-List Control

Characteristic	Intervention Group, No. (%) (n = 136)	Wait-List Control Group, No. (%) (n = 135)
Age, mean (SD), y		
Women	42.0 (8.7)	41.5 (9.3)
Intimate partner	47.5 (9.5)	47.2 (9.6)
Educational level		
≤6 y	25 (19)	15 (11)
7-13 y	101 (75)	111 (83)
Tertiary	9 (7)	8 (6)
Place of birth		
Hong Kong	27 (20)	29 (21)
Mainland China	109 (80)	106 (79)
Duration of marriage, mean (SD), y	21.3 (23.4)	20.2 (21.2)
Marital status		
Married or cohabiting	124 (91)	128 (95)
Single	4 (3)	2 (1)
Divorced	8 (6)	5 (4)
≤1 Child	44 (32)	50 (37)
Chronic illness		
Woman	22 (16)	22 (16)
Intimate partner	16 (12)	18 (13)
Employed		
Woman	34 (25)	21 (16)
Intimate partner	111 (82)	115 (85)
Experiencing financial hardship^a^	93 (69)	93 (69)
Exercises regularly^b^	51 (38)	44 (33)
Smokes daily	6 (4)	4 (3)
Drinks alcohol	8 (6)	7 (5)

^a^ Financial hardship was determined by self-reported financial hardship of the respondent as elicited from the demographic questionnaire.

^b^ Exercising regularly means having a regular exercise routine such as once per week. This information was self-reported.

### Primary Outcome

After 22 weeks, the between-group difference in telomerase activity was not statistically significant (5.18 U [95% CI, 5.05-5.31 U] in the intervention group vs 5.14 U [95% CI, 5.01-5.27 U] in the wait-list control group; *P* = .66; Table 2). When compared with baseline, the increase in telomerase activity in the intervention group after 22 weeks was only marginally significant (*d*, 0.13; 95% CI, 0.001-0.27; *P* = .05), whereas the mean change in the wait-list control group was not statistically significant (*d*, −0.03; 95% CI, −0.16 to 0.10; *P* = .64). Nevertheless, the between-group difference in the change from baseline did not reach statistical significance (*d*, 0.16; 95% CI, −0.02 to 0.35; *P* = .08).

**Table 2.** Outcomes at Baseline, Posttraining Assessment (After Week 6), and Postintervention Assessment (After Week 22) Among Chinese Women Survivors of Intimate Partner Violence Receiving a Qigong Training Intervention vs Wait-List Control

	Intervention Group (n = 136)			Wait-List Control (n = 135)			Between-Group Difference^a^				*P* Value for Group × Time Interaction Effect
							Each Time Point		Change From Baseline		
	Mean (95% CI)	Within-Group Change From Baseline (95% CI)^a^	*P* Value	Mean (95% CI)	Within-Group Change From Baseline (95% CI)	*P* Value	Mean (95% CI)	*P* Value	Mean (95% CI)	*P* Value	
**Primary Outcome**											
Telomerase activity, U^b^^,^^c^											
Baseline	5.05 (4.94 to 5.16)	NA	NA	5.17 (5.06 to 5.28)	NA	NA	0.12 (−0.28 to 0.03)	.12	NA	NA	.08
Postintervention assessment	5.18 (5.05 to 5.31)	0.13 (0.001 to 0.27)	.05	5.14 (5.01 to 5.27)	−0.03 (−0.16 to 0.10)	.64	0.04 (−0.14 to 0.22)	.66	0.16 (−0.02 to 0.35)	.08	
**Secondary Outcomes**											
TNF, pg/mL^c^											
Baseline	1.55 (1.23 to 1.88)	NA	NA	1.31 (0.98 to 1.63)	NA	NA	0.25 (−0.22 to 0.71)	.29	NA	NA	.25
Postintervention assessment	1.35 (1.01 to 1.70)	−0.20 (−0.42 to 0.02)	.08	1.29 (0.95 to 1.63)	−0.02 (−0.23 to 0.20)	.88	0.07 (−0.41 to 0.54)	.79	−0.18 (−0.49 to 0.13)	.25	
IL-6, pg/mL^c^											
Baseline	0.25 (0.07 to 0.42)	NA	NA	0.12 (−0.06 to 0.30)	NA	NA	0.13 (−0.12 to 0.38)	.32	NA	NA	.27
Postintervention assessment	0.12 (−0.07 to 0.30)	−0.13 (−0.30 to 0.04)	.13	0.13 (−0.06 to 0.31)	0.005 (−0.16 to 0.17)	.95	−0.008 (−0.27 to 0.25)	.95	−0.14 (−0.37 to 0.10)	.27	
PSS score^d^											
Baseline	20.65 (19.61 to 21.68)	NA	NA	20.18 (19.14 to 21.22)	NA	NA	0.47 (−1.00 to 1.94)	.53	NA	NA	.008
Posttraining assessment	17.76 (16.72 to 18.81)	−2.88 (−4.13 to −1.64)	<.001	19.57 (18.55 to 20.60)	−0.61 (−1.83 to 0.62)	.70	−1.81 (−3.27 to −0.34)	.02	−2.28 (−3.71 to −0.85)	.002	
Postintervention assessment	17.59 (16.54 to 18.64)	−3.06 (−4.32 to −1.79)	<.001	18.62 (17.60 to 19.65)	−1.55 (−2.79 to −0.31)	.008	−1.03 (−2.50 to 0.43)	.17	−1.50 (−2.95 to −0.05)	.04	
BDI-II score^e^											
Baseline	18.87 (16.93 to 20.81)	NA	NA	17.39 (15.44 to 19.33)	NA	NA	1.48 (−1.26 to 4.23)	.29	NA	NA	.001
Posttraining assessment	11.03 (9.12 to 12.94)	−7.84 (−10.11 to −5.57)	<.001	14.60 (12.73 to 16.48)	−2.78 (−5.01 to −0.56)	.008	−3.57 (−6.25 to −0.90)	.009	−5.06(−7.65 to −2.46)	<.001	
Postintervention assessment	10.69 (8.92 to 12.47)	−8.17 (−10.36 to −5.99)	<.001	12.48 (10.74 to 14.21)	−4.91 (−7.06 to −2.76)	<.001	−1.78 (−4.26 to 0.70)	.16	−3.27 (−5.77 to −0.76)	.01	
PCS score^f^											
Baseline	6.64 (6.28 to 7.00)	NA	NA	6.22 (5.86 to 6.58)	NA	NA	0.42 (−0.09 to 0.93)	.11	NA	NA	.20
Posttraining assessment	5.13 (4.70 to 5.57)	−1.51 (−2.10 to −0.91)	<.001	5.27 (4.85 to 5.69)	−0.95 (−1.54 to −0.37)	<.001	−0.14 (−0.74 to 0.47)	.66	−0.56 (−1.24 to 0.13)	.11	
Postintervention assessment	5.24 (4.80 to 5.69)	−1.40 (−2.00 to −0.80)	<.001	5.33 (4.90 to 5.76)	−0.89 (−1.48 to −0.30)	.001	−0.09 (−0.71 to 0.53)	.78	−0.51 (−1.19 to 0.18)	.15	

Abbreviations: BDI-II, Beck Depression Inventory II; IL-6, interleukin 6; NA, not applicable; PSS, Perceived Stress Scale; PCS, Perceived Coping scale; TNF, tumor necrosis factor.

^a^ Statistics are designated as *d* in descriptions of the study results.

^b^ Percentage relative to a low-telomerase-activity internal control provided by the enzyme-linked immunosorbent assay kit.

^c^ Log transformation was performed.

^d^ The total score range is 0 to 40, higher scores represent higher stress.

^e^ The total score range is 0 to 63, higher scores represent more severe depressive symptoms.

^f^ The total score range is 0 to 13, higher scores represent use of more coping strategies.

### Secondary Outcomes

Regarding proinflammatory cytokines, the concentration of TNF in the intervention group was not significantly different from that in the wait-list control group after 22 weeks (*d*, −0.07 pg/mL, 95% CI, −0.41 to 0.54 pg/mL; *P* = .79). The concentration of IL-6 in the intervention group was also similar to that in the wait-list control group after 22 weeks (*d*, −0.008 pg/mL; 95% CI, −0.27 to 0.25 pg/mL; *P* = .95).

In terms of questionnaire measures on mental well-being, perceived stress (PSS scores) and depressive symptoms (BDI-II scores) were significantly lower in the intervention group than that in the wait-list control group after 6 weeks (PSS: *d*, −1.81; 95% CI, −3.27 to −0.34; *P* = .02; BDI-II: *d*, −3.57; 95% CI, −6.25 to −0.90; *P* = .009) but not after 22 weeks (PSS: *d*, −1.03; 95% CI, −2.50 to 0.43; *P* = .17; BDI-II: *d*, −1.78; 95% CI, −4.26 to 0.70; *P* = .16). Considering the change from baseline, both the PSS and BDI-II scores decreased significantly after 22 weeks in both the intervention group (PSS: *d*, −3.06; 95% CI, −4.32 to −1.79; *P* < .001; BDI-II: *d*, −8.17; 95% CI, −10.36 to −5.99; *P* < .001) and the wait-list control group (PSS: *d*, −1.55; 95% CI, −2.79 to −0.31; *P* = .008; BDI-II: *d*, −4.91; 95% CI, −7.06 to −2.76; *P* < .001). The reduction in both scores was significantly greater in the intervention group than that in the wait-list control group at week 6 (PSS: *d*, −2.28; 95% CI, −3.71 to −0.85; *P* = .002; BDI-II: *d*, −5.06; 95% CI, −7.65 to −2.46, *P* < .001) and week 22 (PSS: *d*, −1.50; 95% CI, −2.95 to −0.05; *P* = .04; BDI-II: *d*, −3.27; 95% CI, −5.77 to −0.76; *P* = .01). For perceived coping (PCS scores), there was no significant between-group difference after 6 weeks (*d*, −0.14; 95% CI, −0.74 to 0.47; *P* = .66) and 22 weeks (*d*, −0.09; 95% CI, −0.71 to 0.53; *P* = .78).

### Feedback From Participants

All intervention participants rated the degree of liking qigong as “like it very much” or “like it.” No wait-list control group participants attended any qigong training throughout the study period.

## Discussion

### Main Findings

To our knowledge, this is the first RCT to evaluate the effects of a qigong intervention on telomerase activity and mental health in Chinese women who have experienced IPV. Telomerase buffers cellular or tissue dysfunction and organismal decline by preventing telomere shortening and enhancing cell viability via extratelomeric functions.^10^ Previous RCTs have shown that mind-body interventions lead to an increase in telomerase activity in healthy adults, dementia caregivers, and individuals with chronic fatigue.^23,26,27^ To our knowledge, this study has the largest sample size (n = 271) relative to previously reported RCTs on telomerase activity. The between-group difference in telomerase activity after 22 weeks did not reach statistical significance, although telomerase activity increased in the intervention group. It is likely that there are other factors affecting telomerase activity including acute stress,^28^ which was not assessed in this study. A recent RCT^29^ investigating the effect of aerobic exercise on telomerase activity of caregivers also found insignificant difference in telomerase activity between the intervention and control groups at the postintervention assessment. Moreover, our study is the first to examine plasma levels of proinflammatory cytokines alongside telomerase activity for exploratory analysis. Again, the intervention did not show significant effect on the levels of TNF and IL-6, which are regarded as important mediators of chronic inflammation. Overall, this study does not support the potential of qigong (a type of moving meditation^13^) to significantly increase the activity of telomerase (an important enzyme beneficial for cell longevity) in Chinese women survivors of IPV.

It is well known that IPV imposes persistent psychological stress on abused women. The findings in this study revealed for the first time that qigong practice results in significantly greater reductions in perceived stress and depressive symptoms in Chinese women survivors of IPV compared with a wait-list control group. Of note, the reduction in BDI-II scores in the intervention group after 22 weeks was clinically meaningful (ie, a change of at least 5 U in BDI-II score) and represented the shift from mild depression to minimal depression.^30^ The beneficial effects of qigong practice are in line with the results of 3 systematic reviews supporting qigong’s beneficial effects against depression.^14,16,31^ As a possible psychological mechanism, qigong may enhance the self-efficacy of participants and hence reduce their depressive symptoms.^32^ As for neurobiological mechanisms, 3 pathways have been suggested: upregulation of monoamine neurotransmitters, stimulation of the hypothalamic-pituitary-adrenal axis, and upregulation of brain-derived neurotropic factors.^33^ Nevertheless, to our knowledge, these mechanisms have yet to be tested. In addition, it is not clear how to explain the improvements in mental health in the wait-list control group. The IPV screening itself may have had some positive influence on mental health.^34^ Furthermore, women in the wait-list control group may have received treatment or services for improving mental well-being outside the study without our knowledge.

### Limitations

Among the numerous studies on interventions in the population of abused women, ours is the first, to our knowledge, to examine the effects at the cellular level. Nonetheless, this study has a few limitations. First, self-reports for measuring psychological outcomes may bias the responses of participants. Second, psychological and physiological mechanisms responsible for the decreased perceived stress and depression in individuals practicing qigong were not examined. Third, the participants were recruited by convenient sampling in Hong Kong; this approach limits generalizability of the findings. Fourth, the long-term benefits of the intervention were not investigated.

### Implications

Despite the insignificant findings on telomerase activity, recommendations can be made for further research. First, more evidence is needed to confirm whether the intervention effect on telomerase activity may have been obscured by variables such as acute stress.^28^ Second, self-reported outcome measures on mental health should be corroborated with physiological measures or objective records. Third, longer follow-up periods are recommended to study the long-term effects of qigong. In terms of clinical application, our approach may offer a safe and low-cost intervention for reducing perceived stress and depressive symptoms in Chinese women survivors of IPV.

## Conclusions

This RCT shows no significant benefit of qigong on telomerase activity in Chinese women survivors of IPV. However, qigong seems to have other benefits regarding perceived stress and depressive symptoms. Further studies are needed to objectively measure the mental health–related outcomes in this population.

## Notes

Supplement 1. **Trial Protocol**

Supplement 2. **Data Sharing Statement**
