# Supplementary material for: Effect of a Qigong Intervention on Telomerase Activity and Mental Health in Chinese Women Survivors of Intimate Partner Violence: A Randomized Clinical Trial
Source: JAMA Netw Open. 2019 Jan 11;2(1):e186967. doi: 10.1001/jamanetworkopen.2018.6967 (PMC6484539; doi:10.1001/jamanetworkopen.2018.6967)
Supplement: Supplement 1. — Trial Protocol [file jamanetwopen-2-e186967-s001.pdf]

Submission to Institutional Review Board

STUDY PROTOCOL

A Randomized, Wait-list Controlled Trial of a Qigong Intervention Program on Telomerase Activity and Psychological Stress in Abused Chinese Women

Principle investigator:
Prof. Agnes Tiwari
Professor and Head
School of Nursing
The University of Hong Kong

Co-investigators:
Prof. Cecilia Lai Wan Chan
Professor and Head
Department of Social Work and Social Administration
The University of Hong Kong

Dr. Rainbow Ho Tin Hung
Assistant Professor
Department of Social Work and Social Administration
The University of Hong Kong

Prof. George Tsao Sai Wah
Professor and Head
Department of Anatomy
The University of Hong Kong

Dr. Wen Deng
Research Assistant Professor
School of Nursing
The University of Hong Kong

Dr. Athena Hong Wai Lin
Teaching Consultant
School of Nursing
The University of Hong Kong

Dr. Daniel Fong Yee Tak
Associate Professor
School of Nursing
The University of Hong Kong

Mrs. Helina Yuk Fung Yin King
Director
H.K.S.K.H Lady MacLehose Centre

Prof. Joyce Ma Lai Chong
Chairperson and Professor
Department of Social Work
The Chinese University of Hong Kong

Table of Content

I. Background Information	2
II. Aims	4
III. Study design	4
IV. Setting	4
V. Participants	5
VI. Intervention	5
VII. Instruments	6
VIII. Measurement	8
IX. Procedures	8
X. Data analysis	9
XI. Ethical approval	10
XII. Potential hazards to participants	10
XIII. Signature of investigators	12
XIV. References	13

I. Background Information

Intimate partner violence (IPV) is a serious, pervasive global public health problem [1]. There is overwhelming evidence that IPV is linked to adverse health outcomes [2]. In Hong Kong, the prevalence of IPV against women [3] has been shown to be comparable to that reported in the World Health Organization's study on violence and health [1] and abused Chinese women also reported significantly more adverse mental health outcomes compared to never abused Chinese women [4].

Globally and particularly in developed countries, numerous interventions have been developed, implemented and evaluated in order to reduce the adverse impact of IPV on survivors' health. Systematic reviews, however, have not found conclusive evidence that the interventions have beneficial effects on abused women's health [5].

To a large extent, protecting abused women's health through efficacious interventions may have been hampered by the lack of a clear understanding about the mechanisms linking IPV to adverse health outcomes. Current literature on lifestyle, well-being and disease risks may help to explain the association between IPV and greater morbidity. Specifically, psychological stress has been shown to be associated with disease risks [6]. As IPV has long been recognized as a potent source of psychological stress [7], the link between IPV and deleterious health outcomes may be related to the chronic psychological stress that abused women often endure as they live in an environment of fear, intimidation and shame. Furthermore, emerging research findings suggest that there are links between psychological stress, risks for disease, and accelerated cellular aging [6]. More recently, a pioneer study investigating cellular aging in abused women found accelerated telomere shortening, a cellular marker of biological aging, in women with a history of IPV [8].

Telomeres are DNA-protein complexes that cap and protect chromosomal ends from deterioration or fusion, and thus play an essential role in maintaining chromosomal stability [9]. It has been shown that exposure to chronic psychological stress hastens shortening of telomeres [10]. When telomeres shorten to a critical point, cells can stop dividing and even become malfunctional [6]. Furthermore, telomere shortening has been shown to be associated with cardiovascular disease, diabetes, dementia, and cancer [6].

It has been demonstrated that biochemical parameters modulate telomere lengths. Specifically, telomerase, an enzyme that counters telomere shortening and adds telomeric DNA

to shortened telomeres, promotes cell longevity even when telomeres are shortened to a critical point [11]. Conversely, dampened telomerase activity could lower the ability to counter telomere shortening [12]. It is thought that the interaction between short telomeres and low telomerase activity may increase the risk of cell death [9]. In addition, telomerase has other telomere-independent functions, such as protecting cells from apoptosis and increasing DNA damage repair, which are important for cell survival and stress resistance. Telomerase activation in human T-lymphocytes has been shown to increase proliferative potential, and, importantly, enhance antiviral activity. Pro-inflammation cytokines (such as TNF-alpha and IL-6) are biochemical modulators of telomerase activity and are closely related to chronic psychological stress. Previous studies have suggested that telomerase activity may be improved through health-promoting behaviors, for example, intensive meditation training [13] and comprehensive lifestyle changes [14]. As detectable changes in telomere length are unlikely to be detected over a short period of time, testing telomerase activity has been suggested as an optimal alternative [14].

Earlier, members of our research team (Chan, Ho) conducted a randomized controlled trial to evaluate the effects of qigong exercise in persons with chronic fatigue. It was found that, among the outcomes, telomerase activity significantly increased in the qigong group when compared to the control group [15]. Qigong is a mind-body exercise rooted in the paradigm of traditional Chinese medicine. It aims to achieve a harmonious flow of energy (*qi*) in the body through gentle movements and is thus considered as a holistic health practice towards promoting physical and mental well-being and improving longevity. In a comprehensive review of the health benefits of qigong, improvement in psychological symptoms was demonstrated [16]. In addition, significant decrease in stress-related biomarkers was also found in the qigong group compared to the control group [17].

Although a link between IPV exposure, duration of IPV-related stress, and telomere shortening was found in an earlier study [8], there is no report yet on how to buffer the effects of psychological stress on cellular aging in abused women. It has been suggested that helping people to adopt certain health-promoting behaviors could alleviate telomere shortening by buffering the effects of chronic psychological stress on cellular aging. Indeed, pilot studies suggest that even short-duration of stress-reduction interventions may slow down or even reverse telomere attrition by increasing telomerase activity [6]. However, none of the

propositions have been tested in abused women. Therefore, the purpose of this proposed randomized, wait-list controlled trial is to evaluate the effects of qigong, a form of health-promoting behavior, on telomerase activity and psychological stress in abused Chinese women.

II. Aims

The primary aim of this randomized, wait-list controlled trial is to evaluate the effect of a qigong intervention program on telomerase activity in Chinese women with a history of intimate partner violence. The secondary aim of this trial is to evaluate the effect of the qigong intervention program on participants' pro-inflammation cytokines, perceived stress, perceived coping, depressive symptoms, and sleep problems.

III. Study Design

A randomized, wait-list controlled design will be used. There will be two groups: an intervention group which will receive an immediate treatment condition (a qigong intervention program) and a wait-list control group which will receive a wait-list control condition (the same qigong intervention program but after the intervention group has completed the treatment condition). Thus, the randomized, wait-list controlled design allows all study participants to eventually receive the intervention but at the same time permits the control of the variables that could cause spurious causality. The justification for using a wait-list control group in this study is that the promise of qigong training can act as an incentive for the control group to participate in the study as well as reduce the risk of treatment contamination.

IV. Setting

The proposed study will be conducted in the catchment areas of the HKSKH Lady MacLehose Centre, which include the districts of Tsuen Wan, Tsing Yi, and Kwai Chung. Over the years, the Centre and the University of Hong Kong School of Nursing have successfully collaborated in a number of community-based services and research activities, which have culminated in the transfer of knowledge to the community for improving the conditions of vulnerable and often impovished recipients.

V. Participants

240 women will be recruited and randomly assigned to either the intervention group (treatment condition, n=120) or a wait-list control group (control condition, n=120). Chinese women are eligible to participate if they are:

- between 18 – 55 years old,
- willing to undertake the qigong intervention program,
- available for all testing points,
- receptive to random allocation, and
- assessed to be abused by an intimate partner in the preceding year or longer, based on the Chinese Abuse Assessment Screen (AAS) (Attachment 1).

We shall exclude the women if they:

- had participated in qigong training within the previous 6 months, or
- have serious medical conditions that might limit their participation in qigong exercise (based on our previous experience, such conditions include cancer, severe obesity, narcolepsy, major depressive disorder, schizophrenia), or
- are abused by someone who is not their intimate partner.

VI. Intervention

The 5.5-month qigong intervention program is based on the intervention that was tested by our team members (Chan & Ho) in an earlier study involving participants with chronic fatigue and achieved positive outcomes [15]. In this proposed project, the qigong intervention consists of:

- <u>Group learning and practice</u>: a 2-hour qigong exercise training session will be provided twice a week for six consecutive weeks (*24 hours*),
- <u>Weekly follow-up</u>: a 1-hour qigong exercise will be conducted with reinforcement of learning and remedial teaching by a qigong master once a week for four consecutive months (16 hours) after the group learning and practice, and
- <u>Self-practice</u>: participant will engage in qigong exercise for 30 minutes every day for

the whole intervention period lasting 5.5 months (63 hours).

For each of the 2-hour qigong exercise training sessions, a breakdown of the activities is as follows:

- A brief introduction to the basic theories of traditional Chinese medicine or educational session on the physiology of mind-body connections (*30-40 minutes*),
- Gentle movement or body stretching in standing postures to facilitate a harmonious flow of *qi* along the energy channels (*20 minutes*), and
- Qigong exercise training delivered by an experienced Daoist qigong master (*1 hour*).

VII. Instruments

The following study instruments will be administered at 3 time points: (a) pre-intervention (T0, baseline), i.e., on entry to study after randomization but before intervention; (b) post-training (T1, 5 weeks later), i.e., on completion of the 5-week qigong exercise training; and (c) post-intervention (T2, 4 months later), i.e., on completion of the 4-month qigong intervention program (with the exception of the Chinese Abuse Assessment Screen and the Demographic Questionnaire, both of which will only be administered at baseline).

(a). The 5-item Chinese version of the Abuse Assessment Screen (AAS) (Attachment 1), which has demonstrated satisfactory psychometric properties [18], will be used to screen potential participants for IPV. If a woman answers "yes" to being emotionally, physically, or sexually abused in the preceding year and if the perpetrator is her former or current intimate partner, she is considered as screened positive for IPV.

(b) The Chinese version of the Revised Conflict Tactics Scales (CTS2) (Attachment 2), which has been validated with satisfactory validity and reliability [3], will be used to measure the type and frequency of behaviors used by the perpetrator during partner conflict. The 27-item instrument consists of 8 measures of pscyhological aggression, 12 measures of physical assault, and 7 measures of sexual coercion. How often each behavior occurred in the preceding

year is indicated by 0=not in past year, 1=once, 2=twice, 3=3-5 times, 4=6-10 times, 5=11-20 times, and 6=21 or more times.

(c) The 10-item Perceived Stress Scale (PSS) (Attachment 3), the most widely used psychological instrument for measuring the perception of stress [19], will be used to assess the degree to which situations in life are perceived by the participant as stressful during the past month. Consisitng of six negative and four positive items, the scores of the PSS are obtained by reversing responses to the four positive items (i.e. 0 [never] =4, 1 [almost never] =3, 2 [sometimes] =2, 3 [fairly often] =1, and 4 [very often] =0) and then sum up across all scale items. The PSS scores may range from 0 to 40.

(d) The Perceived Coping Scale (PCS) (Attachment 4) [20] will be used to assess the types and perceived effectiveness of each of the 13 specific strategies used by the participant in dealing with violence perpetrated by her intimate partner. The strategies may be classified as active or passive including, for example, seeking assistance from the family or friends; confronting the partner; redefining the meaning of the situation; and the use of alcohol and drugs. Scoring is based on a 4-point Likert scale ranging from 1 (not at all helpful), 2 (not so helpful), 3 (somewhat helpful) to 4 (very helpful).

(e) The Chinese version of the Beck Depression Inventory version II (BDI-II) (Attachment 5) will be used to assess depressive symptoms in the previous two weeks. The 21-item instrument has established construct validity and reliability for Chinese populations [21]. Scores may range from 0 to 63, with 0 through 13 indicating minimal depressive symptoms, 14 through 19 mild depressive symptoms, 20 through 28 moderate depressive symptoms, and 29 through 63 severe depressive symptoms.

(f). A Demographic Questionnaire (DQ) (Attachment 6) will be used to elicit the following information: age (participant and her partner), education level, place of birth, number of years living in Hong Kong, marital status, duration of the abusive relationship, number of children, employment status (participant and her partner), financial hardship, receipt of comprehensive social security assistance, need for financial support, and history of chronic illness.

(g) The Chinese version of the General Sleep Disturbance Scale (GSDS) (Attachment 9) is a

21-item questionnaire designed to measure participants' sleep disturbance. Participants will have to rate the frequency of sleep problems during the past week from 0 (not at all) to 7 (every day) [22].

VIII. Measurement

In this proposed study, for each participant, 10 ml of peripheral blood will be collected for measurements of telomerase activity, TNF-alpha and IL-6 levels, at two time points: pre-intervention (T0) and post-intervention (T2) in the intervention group, as well as in wait-list control group before qigong training. The peripheral blood mononuclear cells are isolated for measurements of telomerase activity. Telomerase activity will be analyzed by using a commercially available TeloTAGGG telomerase PCR ELISA (enzyme-linked immunosorbent assay) kit (Roche) [15]. The peripheral blood plasma will be analyzed for pro-inflammation cytokines TNF-alpha and IL-6 levels using commercially available ELISA kits (R&D Systems).

IX. Procedures

Notices will be posted on notice boards and newsletters of the host community centre and its outreach sites. For each of the potential participants, a face-to-face meeting will be arranged in a private area without the presence of her partner. Our trained research assistant will explain the study to the woman, including the need for obtaining blood specimens from her and the nature of the randomized, wait-list controlled trial. If she agrees, a written consent form (Attachment 7) will be signed. Following consent, the AAS will be administered. Women screened as "not abused" by the AAS will be thanked for their participation and no further contact will be made.

Women screened to be "abused" will be randomized to either the intervention or a wait-list control group, according to a list of random permutations prepared by blocked randomization performed by a person not involved in subject recruitment. The block size will be kept securely by the randomizer. The order of allocation will not be altered and will be

centrally controlled in order to avoid any bias in selection. The research assistants will call the central-control centre to obtain the list of random permutations for the allocation.

At entry to study (T0, baseline), women in both groups will be asked to complete the CTS2, PSS, PCS, BDI-II, GSDS, and DQ. Blood sample will be collected from each of the participants by a research nurse trained for this purpose. Following this, the Intervention group will receive the 4-month qigong intervention program. To monitor the self-practice of qigong by participants in the intervention group, they are required to record the frequency and duration of the self-practice of qigong at home and submit the record at the end of the 4-month intervention. Participants in the wait-list control group are advised not to join any outside qigong training class as qigong exercsie training will be provided to them after the final outcome measurement.

At the point of post-training and post-intervention for the intervention group (T1 and T2 respectively), participants in both groups will complete the CTS2, PSS, PCS, BDI-II, and GSDS. Further, blood specimens will also be collected from both groups at T2. Once the intervention group has completed the qigong intervention program, participants in the wait-list control group will receive the qigong exercsie training.

A flow diagram detailing the immediate treatment received by the intervention group and the wait-list control condition received by the control group as well as the data collection points is shown in Attachment 8. We shall provide incentives to the participants in the form of food coupons to show our appreciation of their time and efforts.

X. Data analysis

The primary outcome measure is telomerase activity, while the secondary outcome measures are pro-inflammation cytokines, perceived stress, perceived coping, and depressive symptoms.

Data analysis will be performed using the Statistical Analysis System (SAS). Each estimated effect will be accompanied by a 95% confidence interval, where appropriate. Unless otherwise specified, a 5% level of significance will be used. All statistical methods will be checked for their validity before interpretations are made.

Baseline characteristics (i.e. T0) between the intervention and wait-list control groups will be assessed by chi-square test and Mann-Whitney U test for categorical and continuous data, respectively.

Primary analysis: The telomerase activity at 4 months (i.e. T2) between the intervention and wait-list control groups will be assessed by a regression analysis with adjustment of baseline values. Residuals will be checked to ensure the adequacy of the method. In addition, the change of telomerase activity from baseline (T0) to post-intervention (T2) will be assessed by paired t-test. The intention-to-treat principle will be adopted and all study subjects will be included in the analysis with missing values replaced by the last observed values.

Secondary analysis: Levels of pro-inflammation cytokines (TNF-alpha and IL-6) and the scores of PSS, PCS, BDI-II, and GSDS will be compared for their differences between the intervention group and the wait-list control group by a linear mixed effects model with the baseline value of the scale and intervention group as fixed factor and intercept as random factor. Moreover, effects of demographics on the outcomes will be explored by considering them as fixed factors in the linear mixed effects model. In addition, the change from baseline will also be assessed by a linear mixed effects model with the use of linear contrasts.

XI. Ethical approval

The protocol will be submitted to the Institutional Review Board of the University of Hong Kong/ Hospital Authority Hong Kong West Cluster for approval. The study will be conducted according to the "Declaration of Helsinki". Participation in the study is voluntary. An information sheet is provided and a written consent is required from the participant.

XII. Potential hazards to participants

In our extensive experience of working with abused women, we have found that they generally appreciate telling their abusive experience to professionals who have been trained to receive them. In the event that some women may find the interviewing experience stressful, they will be given the opportunity to have a rest before continuing with the interview. They will also be

offered an opportunity for the social worker to follow them up if necessary. If they choose to withdraw from the study, they may do so with no questions asked.

Blood samples will be taken by a registered nurse who is experienced in venipuncture. If some women feel faint during a venipuncture, the procedure will be stopped. They will be allowed to recline. The procedure will be continued once the individuals recover. If they choose to withdraw from the study, they may do so with no questions asked.

XIII. Signature of investigators

I/We shall comply with the principles enunciated in the 1996 or a later version of the Declaration of Helsinki and HA Investigator's Code of Practice in Undertaking Clinical Research

____

Prof. Agnes Tiwari (Principal Investigator)

____

Prof. Cecilia Lai Wan Chan (Co-investigator)

____

Dr. Rainbow Ho Tin Hung (Co-investigator)

____

Prof. George Tsao Sai Wah (Co-investigator)

____

Dr. Wen Deng (Co-investigator)

____

Dr. Athena Hong Wai Lin (Co-investigator)

____

Dr. Daniel Fong Yee Tak (Co-investigator)

____

Mrs. Helina Yuk Fung Yin King (Co-investigator)

____

Prof. Joyce Ma Lai Chong (Co-investigator)

XIV. References

1. Krug EG, Mercy JA, Dahlberg LL, Zwi AB. The world report on violence and health. Lancet. 2002;360:1083-8.
2. Woods SJ, Gill J. Family violence: Long-term health consequences of trauma. In J. Humphreys, & J. C. Campbell (Eds.), Family violence and nursing practice (2nd edition) (29-50). New York, NY: Springer Publishing Company; 2011.
3. Chan EKL. Study on child abuse and spouse battering: Report on the findings of a household survey. Hong Kong: Department of Social Work & Social Administration, the University of Hong Kong; 2005.
4. Tiwari A, Chan EKL, Fong DYT, Brownridge DA, Lam H, Wong B, Lam CM, Chan F, Chan A, Cheung KB, Ho PC. The impact of psychological abuse by an intimate partner on the mental health of pregnant women. BJOG. 2008;115:377-84.
5. Ramsay J, Carter Y, Davidson L, Dunne D, Eldridge S, Feder G, Hegarty K, Rivas C, Taft A, Warburton A. Advocacy interventions to reduce or eliminate violence and promote the physical and psychosocial well-being of women who experience intimate partner abuse. Cochrane Database Syst Rev. 2009;8:CD005043.
6. Blackburn EH, Epel ES. Too toxic to ignore. Nature. 2012;490:169-71.
7. Woods AB, Page GG, O'Campo P, Pugh LC, Ford D, Campbell JC. The mediation effect of posttraumatic stress disorder symptoms on the relationship of intimate partner violence of IFN-gamma levels. Am J Community Psychol. 2005;36:159-75.
8. Humphreys J, Epel ES, Cooper BA, Lin J, Blackburn EH, Lee KA. Telomere shortening in formerly abused and never abused women. Biol Res Nurs. 2012;14:115-23.
9. Blackburn EH. Telomere states and cells fates. Nature. 2000;408:53-6.
10. Giley D, Herbert BS, Huda N, Tanaka H, Reed T. Factors impacting human telomere homeostasis and aged-related disease. Mech Ageing Dev. 2008;129:27-34.
11. Epel E, Daubenmier J, Moskowitz JT, Folkman S, Blackburn E. Can mediation slow rate of cellular aging? Cognitive stress, mindfulness, and telomeres. Ann N Y Acad Sci. 2009;1172:34-53.
12. Lin J, Epel E, Blackburn E. Telomeres and lifestyle factors: roles in cellular aging. Mutat Res. 2012;730:85-9.
13. Kiecolt-Glaser JK, Glaser R. Psychological stress, telomeres, and telomerase. Brain Behav Immun. 2010;24:529-30.
14. Ornish D, Lin J, Daubenmier J, Weidner G, Epel E, Kemp C, Maqbanua MJ, Marlin R, Yqlecias L, Carroll PR, Blackburn EH. Increased telomerase activity and comprehensive lifestyle changes: a pilot study. Lancet Oncol. 2008;9:1048-57.
15. Ho RTH, Chan JSM, Wang C-W, Lau BWM, So KF, Yuen LP, Sham JST, Chan CLW. A randomized controlled trial of qigong exercise on fatigue symptoms, functioning, and telomerase activity in persons with chronic fatigue or chronic fatigue syndrome. Ann Behav Med. 2012;44:160-70.
16. Jahnke R, Larkey L, Rogers C, Etnier J, Lin F. A comprehensive review of health benefits of qigong and tai chi. Am J Health Promot. 2010;24:e1-e25.
17. Zhang GD. The impacts of 48-form tai chi chuan and yi qi yang fei gong on the serum levels of IgG, IgM, IgA, and IgE in human. Journal of Beijing Institute of Physical Education. 1990;4:12-4.
18. Tiwari A, Fong DYT, Chan EKL, Leung WC, Parker B, Ho PC. Identifying intimate partner violence: comparing the Chinese abuse assessment screen with the Chinese revised conflict tactics scales. BJOG. 2007;114:1065-71.
19. Cohen S, Kamarck T, Mermelstein R. A global measure of perceived stress. J Health Soc Behav. 1983;24:385-96.
20. Yoshihama A. Battered women's coping strategies and psychological distress: Differences by immigration status. Am J Community Psychol. 2002;30:429-52.

21. Byrne BM, Stewart SM, Lee PWH. Validating the Beck Depression Inventory-II for Hong Kong community adolescents. Int J Test. 2004;4:199-216.
22. Lee SY. Validating the General Sleep Disturbance Scale Among Chinese American Parents With Hospitalized Infants. Journal of Transcultural Nursing. 2007;18(2):111-117.
