# Supplementary material for: Effect of a Qigong Intervention on Telomerase Activity and Mental Health in Chinese Women Survivors of Intimate Partner Violence: A Randomized Clinical Trial
Source: JAMA Netw Open. 2019 Jan 11;2(1):e186967. doi: 10.1001/jamanetworkopen.2018.6967 (PMC6484539; doi:10.1001/jamanetworkopen.2018.6967)
Supplement: Supplement 2. — Data Sharing Statement [file jamanetwopen-2-e186967-s002.pdf]

Data Sharing Statement

Cheung. Effect of a Qigong Intervention on Telomerase Activity and Mental Health in Chinese Women Survivors of Intimate Partner Violence. *JAMA Netw Open*. Published January 11, 2019. 10.1001/jamanetworkopen.2018.6967

Data
Data available: Yes
Data types: Deidentified participant data
How to access data: Data are available from the corresponding author on reasonable request. wdeng@hku.hk
When available: With publication

Supporting Documents
Document types: Informed consent form
How to access documents: Informed consent form is available from the corresponding author on reasonable request. wdeng@hku.hk
When available: With publication

Additional Information
Who can access the data: Researchers whose proposed use of the data has been approved and in line with appropriate ethical, data sharing and open access principles
Types of analyses: Analyses which are in line with relevant legislative, research funder and regulatory requirements.
Mechanisms of data availability: After approval of a proposal.
Any additional restrictions: NA
